# Case Report: A case of immune hemolytic anemia after liver transplantation: passenger lymphocyte syndrome is the culprit

**DOI:** 10.3389/frtra.2024.1463325

**Published:** 2024-08-26

**Authors:** Qianzhe Zhao

**Affiliations:** Department of Blood Transfusion, Wuhan Union Hospital, Tongji Medical College, Huazhong University of Science and Technology, Wuhan, China

**Keywords:** immune hemolytic anemia, PLS, post-orthotopic liver transplantation, ABO incompatibility, immunosuppressive therapy

## Abstract

Passenger lymphocyte syndrome (PLS) is most commonly observed after solid organ transplantation with minor ABO blood group incompatibility. It consists of a set of clinical symptoms brought on by the remaining lymphocytes of the donor organ developing antibodies against the recipient's antigens. Here, we describe a typical case of PLS in a type A^+^ recipient receiving a liver transplant from a type O^+^ donor. She suffered from jaundice, abnormally decreased hemoglobin level, and severe hemolytic anemia without bleeding. During hemolysis, we detected a positive direct antiglobulin test (DAT), and the thermal elution test revealed the presence of IgG anti-A antibodies in her serum. When immunosuppressive agents and blood transfusion were used together, cross-matched O^+^ washing red blood cells led to an expected outcome without side effects.

## Introduction

Passenger lymphocyte syndrome (PLS) is an uncommon but serious complication that mainly occurs after solid organ transplantation. Although it is rare among the Kidd, MNS, Kell, and Lewis blood group systems (1–3), it frequently occurs after an ABO or Rh-incompatible transplant, particularly when the recipient is type A (61%) or B (22%) (4). After tissue reperfusion, the recipient's red cell antigen stimulates B lymphocytes from the donor to generate antibodies against the host (5). Intravascular hemolysis is the pattern of manifestation of red blood cell lysis resulting from complement activation (6). The amount of lymphatic tissue transported by the graft, the donor's erythrocyte iso-agglutinin level before transplantation, and the mushroom in antibody titers after transplantation significantly affect the risk of hemolysis (7).

Serological techniques, including DAT, antibody screening and identification, and the elution test, are mainly applied to the diagnosis of PLS. After transplantation, hemoglobin level rapidly falls, and the level of serum bilirubin, lactic dehydrogenase, and other hemolysis-related biochemical indicators increase, which can be used to accelerate diagnosis. The existence of DAT-positive results, anti-recipient erythrocyte antibodies in serum elution test, and the absence of homologous antibodies in antibody screening and identification tests can confirm the diagnosis of PLS (8).

PLS often manifests five to seventeen days after transplantation. While a minority of individuals exhibit severe hemolytic symptoms (9), the majority of patients have self-limited symptoms, with jaundice and anemia as the prominent manifestations. Early ischemia-reperfusion injury, rejection, and other transplantation-related complications can easily be concealed, making timely diagnosis and treatment challenging. Effective symptomatic treatments include inhibiting the activation and proliferation of B cells, elimination of antibodies, and correction of anemia. Transfusions should be conducted with red blood cells that match the donor's ABO type. When hemolysis is severe, plasmapheresis or immunosuppressants can alleviate patients’ symptoms, as there is currently no uniform and dependable method for the diagnosis and treatment of PLS (4). Patients who experience recurrent anemia are more vulnerable to infections, leading to the failure of vital organs and even death. Thus, strengthening the immune system and providing nutritional assistance are of great importance.

## Case report

After suffering from cirrhosis for more than two years, a 56-year-old woman was diagnosed with acute-on-chronic liver failure, which was caused by the decompensation of autoimmune hepatitis after cirrhosis. On April 17, 2024, she underwent a successful allogenic orthotopic piggyback liver transplantation under general anesthesia. With 14 transfusions of A^+^ blood units, the procedure went extremely well. Postoperative vital signs were stable and she was readmitted to the intensive care unit provisionally. Her hemoglobin levels ranged from 70 to 100 g/L 11 days after the surgery. On April 30, 2024, day 13 posttransplant, her hemoglobin level dropped sharply to 46 g/L, but she showed no overt signs of bleeding. At this time, the patient presented with symptoms of acute anemia, such as tachycardia, tachypnea, and hypotension. The physician requested a blood transfusion for hemodilution. The cross-matching procedure indicated that her blood sample was incompatible with several A^+^ donors, thus additional experiments were done. According to preliminary findings, there was a negative antibody screening and a positive DAT with only IgG anti-A antibody in the elution. She needed an emergency blood transfusion to improve her hypoxic symptoms. After receiving 4 units of cross-matched O^+^ washed red blood cells, her hemoglobin level increased to 75 g/L.

**Figure 1:**
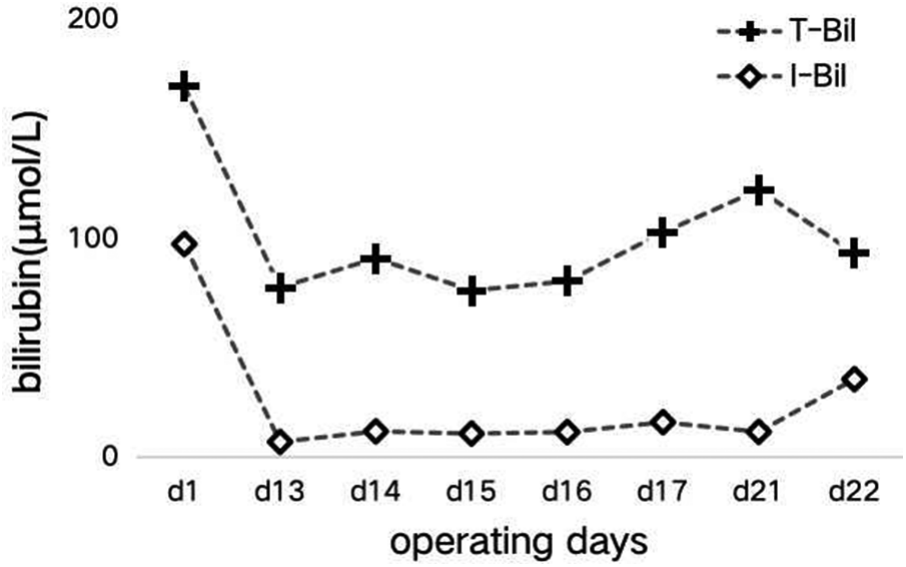
The trend of bilirubin during hospitalization.

**Figure 2:**
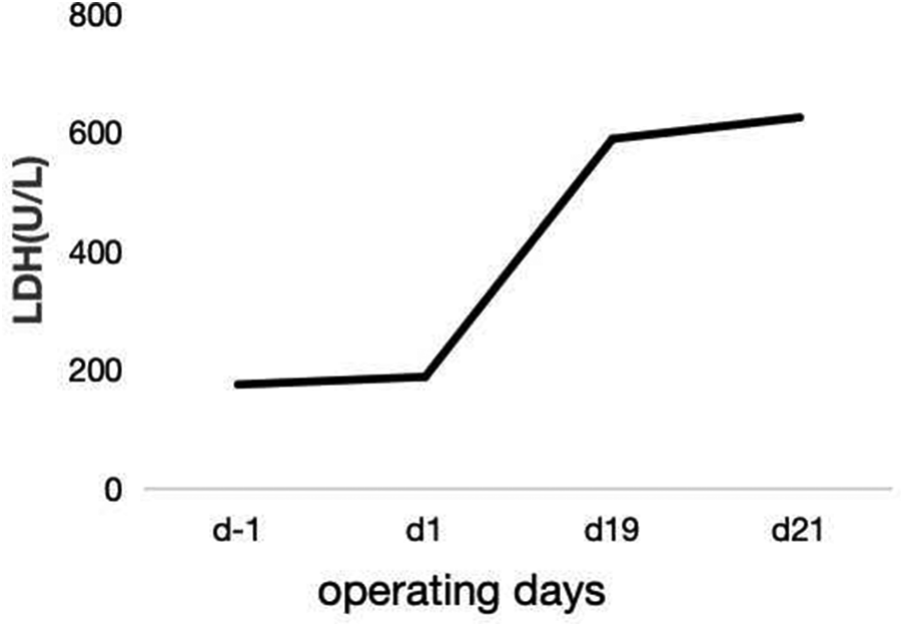
The increase in LDH levels before and after the surgery.

**Figure 3:**
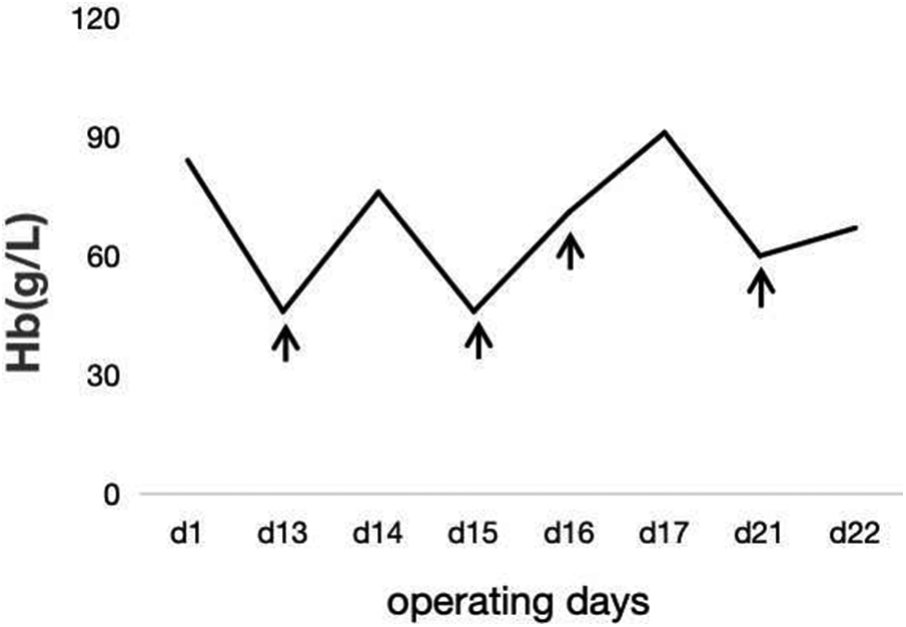
The trend of hemoglobin during hospitalization. The patient's transfusion date is shown in ↑.

In the subsequent inquiry, a week-long laboratory work-up revealed likely hemolysis symptoms, including increased total bilirubin levels of 77.6 μmol/L–122.3 μmol/L (mostly due to increased indirect bilirubin levels) (Figure 1) and lactate dehydrogenase levels of 175 U/L–625 U/L (Figure 2). Based on her recent liver transplantation, these biochemical results, and escalating pale and anemic appearance, we suggested that PLS possibly led to her hemolysis. Her anemia was acute hemolytic anemia, belonging to normochromic normocytic anemia. Her hemoglobin levels were maintained at 70 g/L, and she experienced no adverse reactions by means of receiving four transfusions (Figure 3). In total, until this stage, she received 16 units of O^+^ washed RBCs. In addition, she received hormones, immunoglobulins, and tacrolimus to induce immunosuppression and prevent infection.

## Discussion

PLS typically occurs after hematopoietic stem cell transplantation and solid organ transplantation. It is also the major cause of hemolysis after transplantation. This type of immune hemolysis is most common in cardiopulmonary transplantation (70%), followed by liver and kidney transplantations (29% and 9%, respectively) (7). When the donor's B lymphocytes enter the recipient, they develop antibodies against the recipient’s erythrocyte antigen, which leads to a hemolytic reaction (10). PLS is principally characterized by signs of hemolysis, such as jaundice and anemia. The diagnosis can be made when patients have a positive direct antiglobulin test and eluted anti-recipient erythrocyte antibodies in their serum. The majority of the therapeutic approaches are semiological, such as blood transfusion therapy, blocking B cell activation and proliferation, and antibody elimination.

Our patient suffered a hemoglobin plunge and increased total bilirubin and lactate dehydrogenase levels 13 days after her liver transplantation. The serological tests revealed that DAT was positive. Alloantibodies were excluded using antibody screening and identification assays. Only IgG anti-A antibodies were found in her serum. The serum biochemical indices and typical clinical symptoms of this patient were assessed, which were consistent with the diagnosis of PLS. Early anemia after liver transplantation can be caused by hemorrhage, septicemia, or medications. The cumulative incidence of early anemia ranges from 4.3% to 28.2%. Immune hemolysis, such as that produced by ABO incompatibility and graft-versus-host disease, accounts for less than 1% of the total cases of hemolysis (4). Her hemolytic anemia caused by PLS was symptomatically treated by blood transfusion, and the symptoms of hypoxia significantly improved. Immunosuppressive agents, including 80 mg/qd methylprednisolone sodium succinate and 5 g/Tid human immumoglobulin (PH4) were intravenously injected. Tacrolimus dosage varied from 1 mg/q12 h to 2 mg/q12 h depending on its blood concentration. The vital signs of our patient were stabilized.

As organ transplantation medical technology has matured and become the most efficient treatment for patients with end-stage organ failure, the number of patients who undergo transplantation has gradually increased in recent years. However, due to the rarity of donors, non-identical organ transplants are frequently used as an alternative. The incidence of PLS following liver transplantation is around 30%. According to a study conducted at La Fe University Hospital in Spain, 12 of 1217 individuals who underwent liver transplantation developed PLS after surgery. Among them, 10 patients had ABO-incompatible transplants and 2 had Rh-incompatible transplants. Hemolysis was identified in all patients (11). Nguyen et al. described a case of hemolytic anemia caused by PLS three weeks after liver transplantation. The donor and recipient had both AB blood type, while incompatibility was caused by the RhD antigen. This patient was RhD-negative and had a history of being positive for anti-D antibodies. From their viewpoint, Rh blood types were associated with more severe anemia than ABO blood group-induced hemolysis. The specific reason behind this association is unknown, and the reason may be that Rh antibodies are produced over a longer period (12).

Based on various case reports, we believe that early detection of PLS after transplantation and multimodal symptomatic management are critical to effectively improve the prognosis. Therefore, we recommend routine monitoring of anti-A, anti-B antibodies and DAT, the serum biochemical indices of ABO-incompatible transplant patients from 5 to 17 days after surgery, so as to detect PLS and give symptomatic treatments as soon as possible (5). If ABO or Rh blood groups are non-homotypic or the donor and recipient are minor compatible, the possibility of PLS should be fully predicted and the appropriate treatment plans should be prepared. Tentative non-functional assays, such as the monocyte monolayer assay (MMA), can be identified to differentiate the role of alloantibodies. Similar to antibody identification test, it help to rule out the presence of irregular antibodies other than ABO in patient's serum. For this syndrome tending to happen in the acute phase of post-transplant immunosuppression, doctors must carefully weigh the risks and advantages of immunotherapy. Meanwhile, the blood transfusion department should choose and deliver matching blood in a timely manner after reporting severe life-threatening ischemia and hypoxia. The prudent transfusion of O-type red blood cells after surgery possibly does not reduce the risk of PLS, for the main cause of PLS is unrelated to blood transfusion. In addition, the red blood cell formulations we deliver to patients are treated with white blood cell removal or even irradiation to reduce further immunostimulation to the patient's immune system. These have provided greater help to alleviate the adverse progress of PLS and for doctors to treat patient more comprehensively. If a patient who has been diagnosed with PLS in the past is faced with the need for blood transfusion again, we still recommend to review the DAT and elution test, and according to the principle of compatible blood transfusion, the patient should be transfused with cross-matched blood.

In this case, we witnessed a nearly perfect performance of the liver transplantation team, close attention to the clinical symptoms and vital signs of the patient after the surgery, and timely communication with the blood transfusion department. Laboratory parameters were collected and various serological tests were conducted. These indicators helped doctors to make a timely diagnosis of PLS, take appropriate therapeutic measures, and improve the patient's prognosis. We believe that effective communication and collaboration between the blood transfusion department and the clinical departments can significantly advance organ transplantation. The blood transfusion department shall cooperate with doctors to diagnose PLS in a timely manner and improve the level of technical ability when dealing with diversified postoperative adverse reactions.

## Data Availability

The original contributions presented in the study are included in the article/Supplementary Material, further inquiries can be directed to the corresponding author.
